# Malignant mesothelioma with unexpected contralateral mediastinal shift: a case report

**DOI:** 10.1186/1752-1947-2-125

**Published:** 2008-04-28

**Authors:** James S Myerson, Shibani Nicum, Bhupinder Sharma, Mary ER O'Brien

**Affiliations:** 1 The Royal Marsden NHS Foundation Trust, Downs Road, Sutton, Surrey, SM2 5PT, UK

## Abstract

**Introduction:**

Contralateral mediastinal shift due to pleural mesothelioma tissue, rather than a pleural effusion, is an unusual clinical feature of mesothelioma.

**Case presentation:**

A 63-year-old woman with a past history of treated invasive ductal carcinoma of the breast presented with breathlessness and chest pain. Her chest radiograph revealed contralateral mediastinal shift and drainage of over 3 litres of pleural fluid relieved her symptoms. She underwent further investigations which revealed pleural mesothelioma, rather than the expected metastatic breast cancer. When she represented with breathlessness a few months later, a chest radiograph again demonstrated contralateral mediastinal shift. A thoracic ultrasound on this occasion revealed only a small loculated pleural effusion and, unexpectedly, a large volume of malignant tissue, thereby explaining the chest radiograph appearances.

**Conclusion:**

This case illustrates mediastinal shift away from the affected side which was caused by mesothelioma tissue itself, rather than by a pleural effusion which is the more usual cause of contralateral mediastinal shift in mesothelioma.

## Introduction

In malignant mesothelioma, the hemithorax affected is usually contracted and imaging typically shows ipsilateral volume loss, and sometimes, mediastinal shift towards the affected side. Pleural fluid associated with malignant mesothelioma is a very common presentation of this disease and may occasionally cause mediastinal shift if the effusion is large enough. However, mediastinal shift due to the mesothelioma tissue itself rather than associated fluid, is uncommon and there is only one previously reported case series of four patients 20 years ago [1]. We present the case of a 63-year-old lady with malignant mesothelioma of the left hemithorax.

## Case Presentation

**Figure 1:**
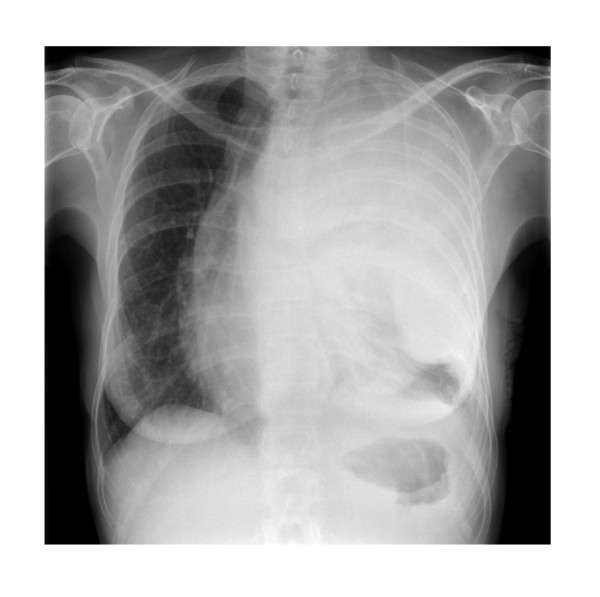
Chest Radiograph, November 2006.

**Figure 2:**
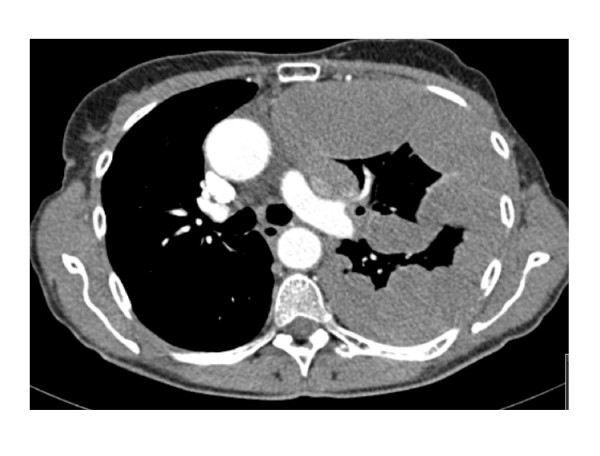
Computer Tomography of Thorax, November 2006.

Our patient, a 63-year-old woman, initially presented in 1992 with a T1N0M0 invasive ductal carcinoma of the left breast, which was treated with quadrantectomy, post-operative radiotherapy and adjuvant tamoxifen for 5 years. She continued under regular follow-up until November 2006 when she developed breathlessness and chest pain. A chest radiograph demonstrated extensive shadowing in the left hemithorax with associated mediastinal shift (figure 1). An intercostal chest drain was inserted and 3080 mls of blood-stained fluid was drained off, relieving her dyspnoea. A CT scan however, performed in the month after pleural drainage, showed much of the hemithorax was occupied by circumferential, predominantly low attenuation, pleurally based thickening in contact with the superior mediastinum, pericardium and great vessels. There was also extrinsic compression of the pulmonary artery to the left upper lobe, and the left atrium was deviated to the right due to an impinging abnormal soft tissue mass (figure 2).

A pleural biopsy performed via a Video Assisted Thoracoscopic Surgery (VATS) procedure revealed a new primary malignant mesothelioma rather than the expected secondary spread from her breast carcinoma.

Following pleural drainage, she received exit site radiotherapy. She subsequently became more short of breath and a chest radiograph indicated that there was persistent mediastinal shift.

**Figure 3:**
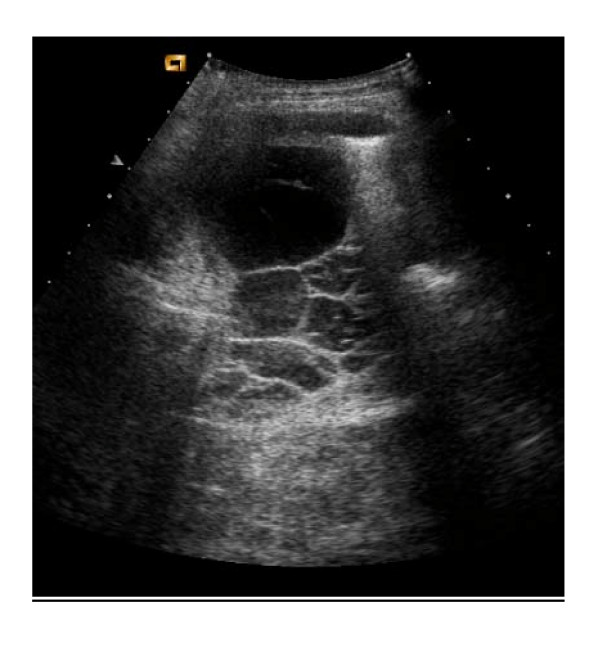
Ultrasound of Thorax, January 2007.

A thoracic ultrasound (figure 3) demonstrated only a small volume of loculated fluid (4 × 8 cm) suggesting that much of the shadowing seen on the chest radiograph was malignant tissue. Her breathlessness was managed with other therapies, including systemic chemotherapy. The breathlessness improved.

## Conclusion

This case illustrates an unusual clinical picture of mesothelioma. We believe it is a useful case to bring to other physicians' attention, as whilst the chest radiograph suggested a large pleural effusion, the CT scan and ultrasound showed this not to be the case. In this situation, repeated attempted pleural drainage would not help the patient's symptoms and may cause unnecessary distress. It shows the effects seen in a patient with mesothelioma with a large burden and volume of disease.

## Abbreviations

CT: Computer Tomography.

## Competing interests

The authors declare that they have no competing interests.

## Authors' contributions

JSM prepared the case report. SN and MERO reviewed and added to the case report. BS prepared the radiology figures. All authors have read and approved the final version of the manuscript.

## Consent

The patient is now deceased. Written informed consent was obtained from the patient's next of kin for publication of the report and any accompanying images. A copy of the written consent is available for review by the Editor-in-Chief of this journal.
